# Humoral, T cell and immune gene expression responses to SARS-CoV-2 vaccination in a small group of children with previous MIS-C

**DOI:** 10.1016/j.vaccine.2025.127461

**Published:** 2025-07-05

**Authors:** Timothy F Spracklen, Jonathan Day, Hamza Van Der Ross, Claire Butters, Ntombi Benede, Avril Walters, Rubina Bunjun, Thandeka Moyo-Gwete, Mashudu Madzivhandila, Simon C Mendelsohn, Thomas J Scriba, Muki Shey, Wendy A Burgers, Penny L Moore, Liesl J Zühlke, Roanne S Keeton, Kate Webb

**Affiliations:** a Department of Paediatrics and Child Health, University of Cape Town, Cape Town, South Africa; b Cape Heart Institute, University of Cape Town, Cape Town, South Africa; c Institute of Infectious Disease and Molecular Medicine, University of Cape Town, South Africa; d Division of Medical Virology, Department of Pathology, University of Cape Town, South Africa; e SAMRC Antibody Immunity Research Unit, School of Pathology, University of the Witwatersrand, Johannesburg, South Africa; f National Institute for Communicable Diseases of the National Health Laboratory Services, Johannesburg, South Africa; g South African Tuberculosis Vaccine Initiative, Division of Immunology, Department of Pathology, University of Cape Town, Cape Town, South Africa; h Department of Medicine, University of Cape Town, Cape Town, South Africa; i CIDRI-Africa, Institute of Infectious Disease and Molecular Medicine, University of Cape Town, South Africa; j Wellcome Centre for Infectious Diseases Research in Africa, University of Cape Town, South Africa; k Centre for the AIDS Programme of Research in South Africa, Durban, South Africa; l South African Medical Research Council, Cape Town, South Africa; m Crick African Network, The Francis Crick Institute, London, United Kingdom

## Abstract

The effects of SARS-CoV-2 vaccination in children with previous multisystem inflammatory syndrome (MIS-C) are not well understood. In this study, we aimed to assess immune responses to SARS-CoV-2 vaccination in children over the age of 12 years with previous MIS-C and compare them to healthy children. Three children with previous MIS-C and four healthy children received two doses of the BNT162b2 vaccine. Blood was collected before the first dose, one week after the first dose, one week after the second dose and three weeks after the second dose. All participants had detectable SARS-CoV-2 spike IgG before vaccination. Spike binding and neutralising antibody activity increased after the first vaccine dose with no differences between children with a history of MIS-C and healthy children. Serum inflammatory cytokines and whole blood immune gene profiles did not resemble acute MIS-C and there were no differences in these between the two groups at any timepoint. All participants gained a robust SARS-CoV-2-specific T cell response by three weeks after the second dose. A transient increase in SARS-CoV-2-specific CD4 T cells expressing TCR Vβ21.3, a non-specific T cell subset previously found to be enriched in patients with MIS-C, was demonstrated in two of the children with previous MIS-C but none of the healthy children. Together, these data demonstrate that vaccination is effective at boosting SARS-CoV-2-specific immune responses in a small group of children with previous MIS-C, and that it does not induce inflammatory cytokine or gene expression responses resembling acute MIS-C. Although larger-scale studies are needed to confirm these findings, the present evidence supports SARS-CoV-2 vaccination in children with previous MIS-C.

## Introduction

Multisystem inflammatory syndrome in children (MIS-C) is a severe immune hyperactivation syndrome associated with SARS-CoV-2 infection in children. MIS-C presents in children two to six weeks after exposure to SARS-CoV-2 with elevated temperatures, rash, conjunctivitis and variable organ involvement including myocarditis and shock.

The exact immune sequence that causes MIS-C is not fully understood, although it is characterised by markedly raised inflammatory markers, a unique inflammatory gene expression signature and significantly elevated inflammatory cytokines [1–8]. Children with acute MIS-C have evidence of a maturing immunoglobulin (Ig)G response to SARS-CoV-2, but no differences in the titres or function of SARS-CoV-2 antibodies compared to children exposed to SARS-CoV-2 who do not develop MIS-C, arguing against an antibody-mediated pathogenesis [1, 9]. An over-expression of non-specific T cells expressing TCR Vβ21.3 has also been reported in patients with MIS-C, which may indicate a non-specific superantigen response [10]. Recent evidence suggests that cross-reactivity between SARS-CoV-2 and human SNX8 protein may underly development of MIS-C [11].

In population studies, SARS-CoV-2 vaccination has been shown to be protective against MIS-C [12–15]. In addition to immunological benefits of SARS-CoV-2 vaccination, there is evidence that this vaccination is safe and well tolerated in children with previous MIS-C [16–20]. The Centers for Disease Control and Prevention (CDC) therefore recommends vaccination of children with previous MIS-C after full clinical recovery and at least 90 days after diagnosis of MIS-C [21]. There are currently no in-depth data describing the inflammatory responses to re-exposure to SARS-CoV-2 antigen through vaccination in children with previous MIS-C. In this study, we aimed to assess the immunogenicity and the inflammatory cytokine, T cell and gene expression responses of SARS-CoV-2 vaccination in a small group of children with previous MIS-C and healthy children.

## Methods

### Patient cohort

Children with previous MIS-C between the ages of 12 and 18 years were offered vaccination on the State vaccination programme, which was only offering vaccination to children over 12 at the time. Other inclusion criteria included no prior SARS-CoV-2 vaccination and previous enrolment in an MIS-C study carried out from June 2020 to March 2022 at the Red Cross War Memorial Children’s Hospital in Cape Town, South Africa [1, 22]. All patients fulfilled the World Health Organisation criteria for MIS-C [23] at the time of their diagnosis and were healthy at the start of this study. Controls were healthy unvaccinated children enrolled in the abovementioned MIS-C study or referred by other participants. Previous COVID-19 was not recorded for the healthy control children as prior SARS-CoV-2 exposure was not a criterion for exclusion. No children had any underlying chronic infectious or immune diseases.

Ethical approval was obtained from the University of Cape Town Human Research Ethics Committee (HREC 599/2020) and informed consent was collected from all participants. Unvaccinated patients with acute MIS-C (n = 30) and contemporaneous unvaccinated healthy controls (n = 74) were included as comparator groups in some of the assays below (serum cytokine and chemokine analysis and gene expression analysis).

### Vaccination schedule and sampling

Children received two doses of the Pfizer-BioNTech COMIRNATY® SARS-CoV-2 mRNA vaccine (BNT162b2) three weeks apart. Longitudinal serum, plasma, whole blood and PAXgene® blood RNA samples were collected before the first dose, one week after the first dose, one week after the second dose and three weeks after the second dose.

### SARS-CoV-2 antibody enzyme-linked immunosorbent assay (ELISA)

Serum from all participants at all four timepoints was assessed to measure anti-SARS-CoV-2 spike IgG antibodies. Briefly, 2 µg/ml of SARS-CoV-2 spike protein was added to each well in a 96-well plate to coat the plate and was incubated overnight at 4°C. After washing, plates were blocked by incubating in blocking buffer (5% skimmed milk, 0.05% Tween 20 and 1X PBS). Samples were diluted to 1:100 and added to plates after they had been washed. The secondary antibody (anti-IgG) was then added at a dilution of 1:3000 in blocking buffer. TMB substrate was added and after 5 minutes, the reaction was stopped using 1M H_2_SO_4_. The absorbance was measured at a wavelength of 450 nm.

### SARS-CoV-2 antibody neutralisation assay

Serum from all participants at all four timepoints was assessed for neutralisation activity. Briefly, HEK293T cells were co-transfected with SARS-CoV-2 spike (D614G) plasmid and a firefly luciferase lentivirus backbone plasmid to create SARS-CoV-2 pseudotyped lentiviruses. Serum samples were heat-inactivated and incubated with the SARS-CoV-2 pseudotyped lentiviruses for 1 hour at 37°C and 5% CO_2_. HEK293T cells over-expressing ACE-2 were added and incubated for 72 hours. Luminescence was measured and ID_50_ values calculated.

### Serum cytokine and chemokine analysis

Serum from all participants at all four timepoints was assessed. A customised MILLIPLEX® kit was used to determine the serum concentration of interleukin (IL)-1 receptor antagonist (RA), IL-1β, IL-10, IL-6, IL-27, monocyte chemoattractant protein (MCP)-1, interferon gamma-induced protein (IP)-10 and tumour necrosis factor (TNF) using a Bio-Plex platform as per manufacturer’s instructions. Briefly, antibody immobilised beads were added to wells containing standards, controls and samples for 2 hours at room temperature. After washing, the detection antibody was added and incubated for 1 hour, whereafter Streptavidin-Phycoerythrin was added for 30 minutes in the dark. The plate was washed and sheath fluid was added to each well, after which the plate was read using a Luminex plate reader. Fold changes were calculated using the geometric means of all participants at the different timepoints.

### RNA extraction and gene expression analysis

Gene expression analysis of 96 individual transcripts was measured from RNA derived from whole blood, using qRT-PCR as previously described [24]. Transcripts included housekeeping genes and other genes with broad relevance to inflammation, immunopathology, immune regulation and type I interferon responses. RNA samples were available for each participant and at each timepoint. Nine healthy control individuals were also included on the chip. Data were combined with our previously described pre-treatment acute MIS-C (n = 30) and healthy (n = 54) gene expression data after adjusting for batch effect in R v4.3.1 with the batchtma package and using the quantreg method. The data were used to assess changes in expression of our previously described 29-gene signature for MIS-C [24], although two of these genes, *MMP9* and *TLR8*, were removed from the analysis due to high failure rates of the probes (67.9% and 42.9% failure rates, respectively).

### T cell flow cytometry

The whole blood assay sample processing used for this study was adapted from a whole blood intracellular cytokine detection assay designed to detect TB-specific T cells in children [25]. Briefly, whole blood was collected in sodium heparin tubes and stimulated with 1 µg/ml SARS-CoV-2 spike peptide pools of 15mer peptides overlapping by 11 amino acids (Miltenyi Biotec) in the presence of costimulatory antibodies against CD28 (clone 28.2) and CD49d (clone L25) (1 μg/mL each; BD Biosciences) and Brefeldin A (10 μg/mL, Sigma-Aldrich). Unstimulated blood was incubated with costimulatory antibodies, Brefeldin A and an equimolar amount of DMSO as a background control. After 24 hours, blood was treated with EDTA (2 mM) for 15 minutes followed by red blood cell lysis and cell fixation using FACS lysing solution (BD Biosciences) for 10 minutes. Cells were then cryopreserved in freezing media (90% fetal bovine serum and 10% DMSO) and stored at −80°C until batched analysis.

Fixed, cryopreserved cells were thawed, washed and surface stained with CD4 ECD (SFCI12T4D11; Beckman Coulter), CD8 BV510 (RPA-8; BioLegend) and TCR Vβ21.3 FITC (REA894; Miltenyi Biotec). Cells were then permeabilised and intracellularly stained with CD3 BV650 (OKT3; BioLegend) and IFN-γ AlexaFluor®700 (B27; BioLegend). Samples were acquired on a BD LSR-II Fortessa flow cytometer (BD Biosciences) using Diva software (v9) and analysed using FlowJo (v10). Cells were gated on singlets, lymphocytes and CD3 cells. Results are expressed as the frequency of CD4 or CD8 T cells expressing IFN-γ or the frequency of SARS-CoV-2-specific CD4 T cells expressing TCR Vβ21.3. Cytokine responses are presented as background subtracted values (from the frequency of cytokine produced by unstimulated cells).

### Statistical analysis

Analyses of cytokines and gene expression were conducted using R. Pairwise differences were assessed for each timepoint using two-tailed Mann Whitney U tests, corrected for multiple comparisons using the Holm-Bonferroni method. The pheatmap package was used to perform hierarchical clustering of the gene expression data with centering and scaling of variables. Prior to principal component analysis (PCA), the missForest package was used for random-forest-based imputation of missing delta Ct values with 100 trees in each forest [26]. PCA was performed using the base R prcomp function with zero centering and scaling of variables and visualised with the ggbiplot package.

Statistical analysis of T cell responses was conducted in GraphPad Prism. Due to the small sample size, statistical analysis was limited to a nonparametric Mann Whitney U test to detect differences in SARS-CoV-2-specific CD4 T cell responses between healthy and MIS-C participants at the final timepoint at which all participants had SARS-CoV-2-specific CD4 T cell responses, and a paired Wilcoxon signed rank analysis of the total TCR Vβ21.3 expressing CD4 and CD8 T cells at the third-last timepoint compared to the pre-vaccination timepoint.

## Results

### Vaccination cohort

**Figure 1:**
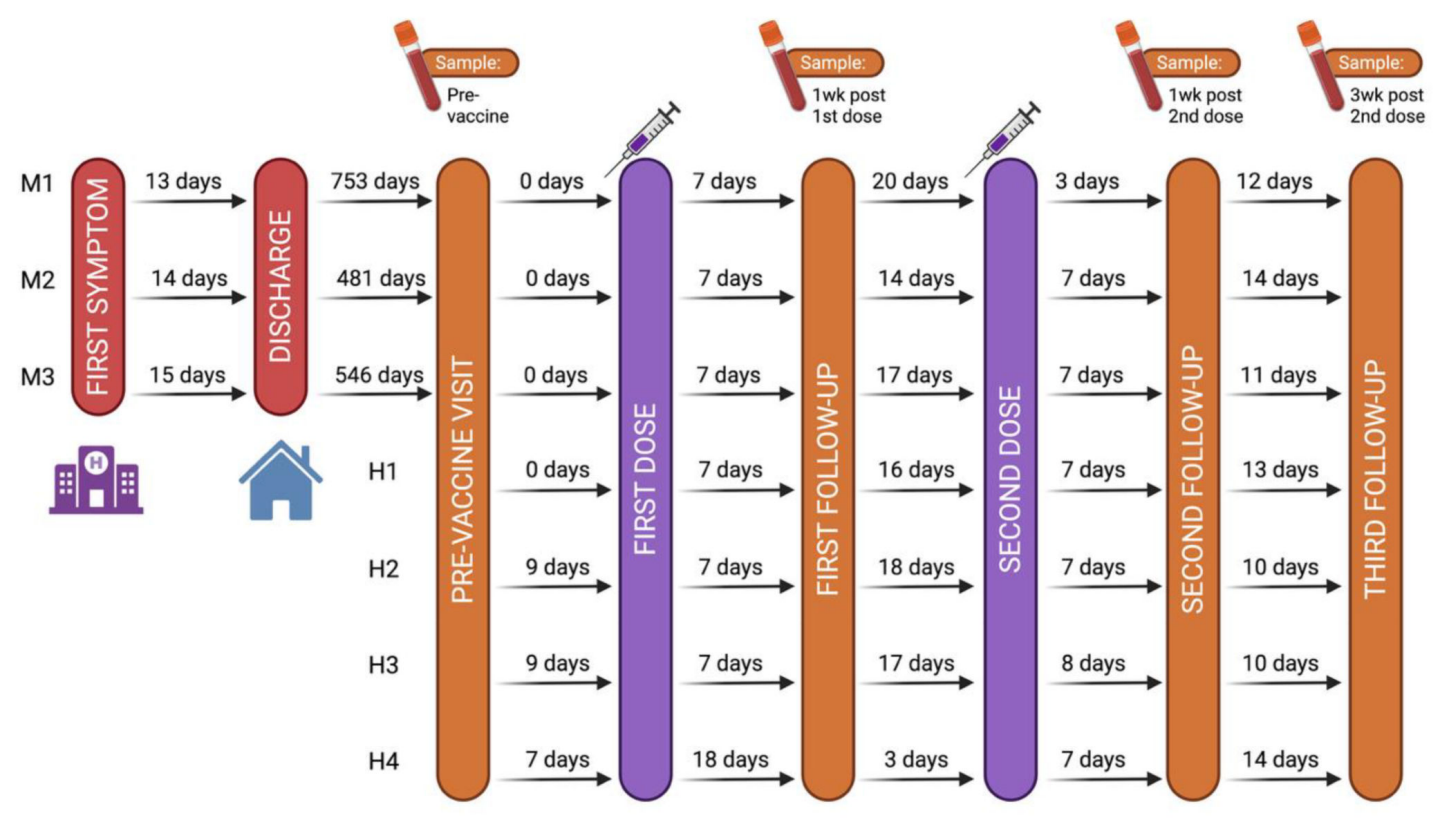
Study design for vaccination of children with previous MIS-C and healthy controls. Participants M1, M2 and M3 were children with previous MIS-C, while H1, H2, H3 and H4 were healthy participants.

Of the 66 patients with a history of MIS-C who were previously enrolled, 15 children were over the age of 12 years. Of these, 10 were successfully contacted and 3 agreed to be enrolled in the study. Of the 110 healthy controls who were previously enrolled, 20 were over the age of 12 years. Two consented to be enrolled, and an additional two children over 12 years were referred by one of the participants. These seven children were vaccinated between July and September 2022 (Figure 1). All participants were vaccinated between the ages of 12 and 13 years (Supplementary Table S1).

Of the three previous MIS-C patients, one was diagnosed with MIS-C during the first epidemiological SARS-CoV-2 wave in South Africa, while the other two were diagnosed in the second SARS-CoV-2 wave dominated by the Beta variant (Supplementary Table S2). Both patients recruited in the second wave required multiple intensive care unit (ICU) stays during their hospital admission. All patients clinically recovered and were discharged within 10-11 days. The clinical courses of their diseases are summarised in Supplementary Table S2. All patients presented with tachycardia, and all had fever, lung disease and gastrointestinal involvement at some time during their admission. All patients had full resolution of all symptoms on follow up and were clinically well at time of vaccination.

### Vaccine-induced SARS-CoV-2 spike-antibody titres are not affected by a history of MIS-C

**Figure 2:**
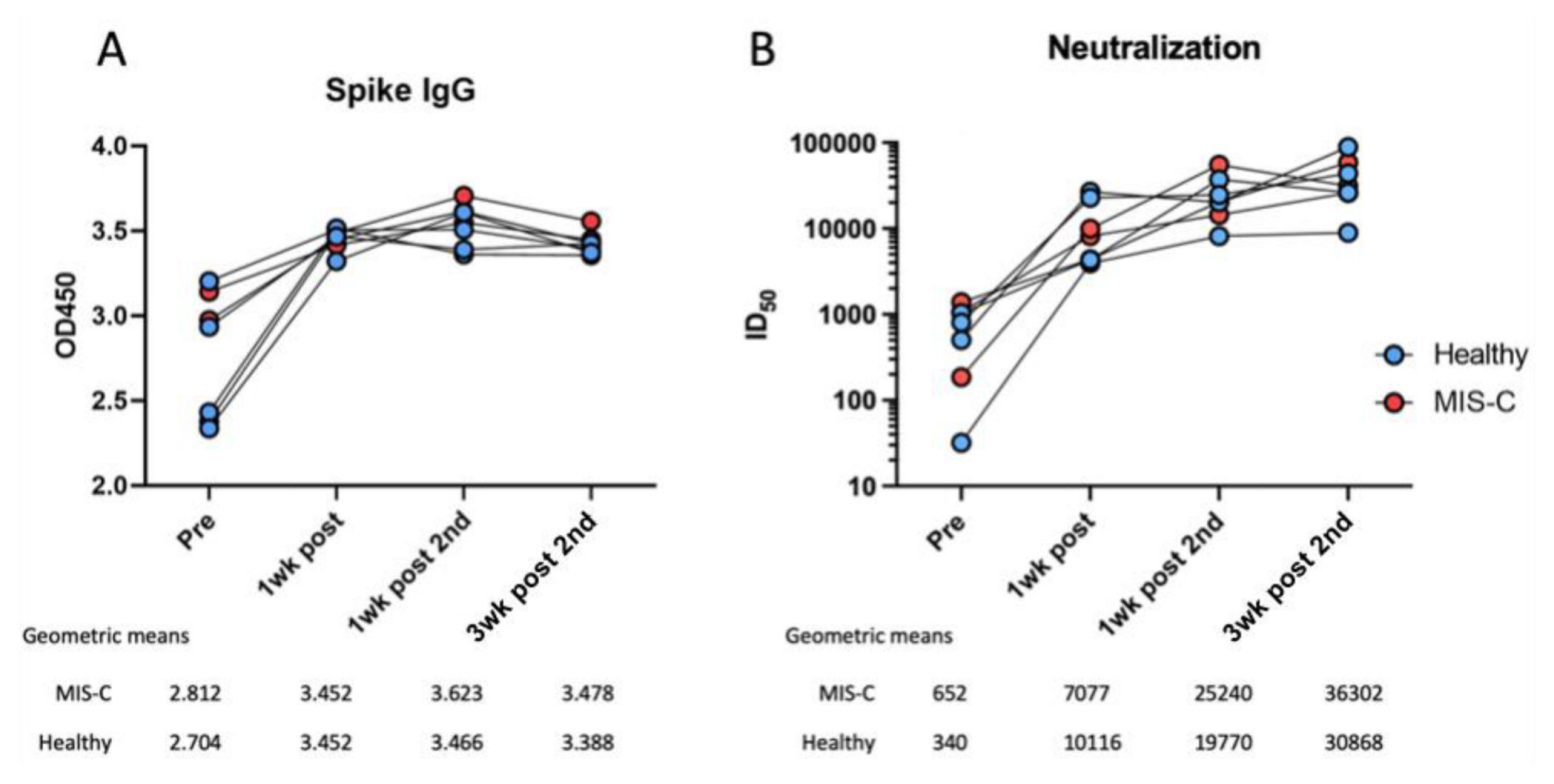
Anti-SARS-CoV-2 antibodies in serum of vaccine recipients. (A) Anti-spike IgG antibody levels measured at a dilution of 1:100 in the serum of participants with a history of MIS-C (red) and healthy participants (blue) before receiving a dose of the SARS-CoV-2 vaccine, one week after the first dose of the vaccine, one week after the second dose of the vaccine and three weeks after the second dose of the vaccine. (B) Neutralisation ID_50_ values of anti-spike protein antibodies in the participants across the vaccination schedule. Note that some individual values may be very similar: for individual datapoints please refer to Supplementary Table S3.

Humoral responses to SARS-CoV-2 vaccination were determined by measuring SARS-CoV-2 spike IgG antibody levels and neutralising abilities in all participants at all timepoints. Before vaccination, all seven participants had positive (OD450 > 0.4) SARS-CoV-2 spike-antibody ELISA values (Figure 2A), indicating prior exposure to SARS-CoV-2. Following the first vaccine dose, there was a noticeable increase (1.26-fold) in the SARS-CoV-2 spike-antibody responses in both groups, with no difference between the two groups at any timepoint. The responses remained the same after the first dose, with no detectable boost after the second dose and no appreciable waning in antibody titres (1.26-fold difference between pre-vaccine titres and the final timepoint). After the second dose, the assay reached saturation, and the data were inconclusive as to whether the second dose increased antibody titres or showed waning by six weeks.

The fully titrated neutralising ability of the SARS-CoV-2 spike-antibodies increased 37-fold in both cohorts following the first vaccine dose with no difference between children with a history of MIS-C and healthy children at any of the four timepoints (Figure 2B). There was no evidence of waning neutralising ability, but there was a 99-fold increase in neutralising ability following the second SARS-CoV-2 vaccine dose and a 106-fold increase at the final timepoint.

### Cytokine production was not increased with vaccination in children with previous MIS-C

**Figure 3:**
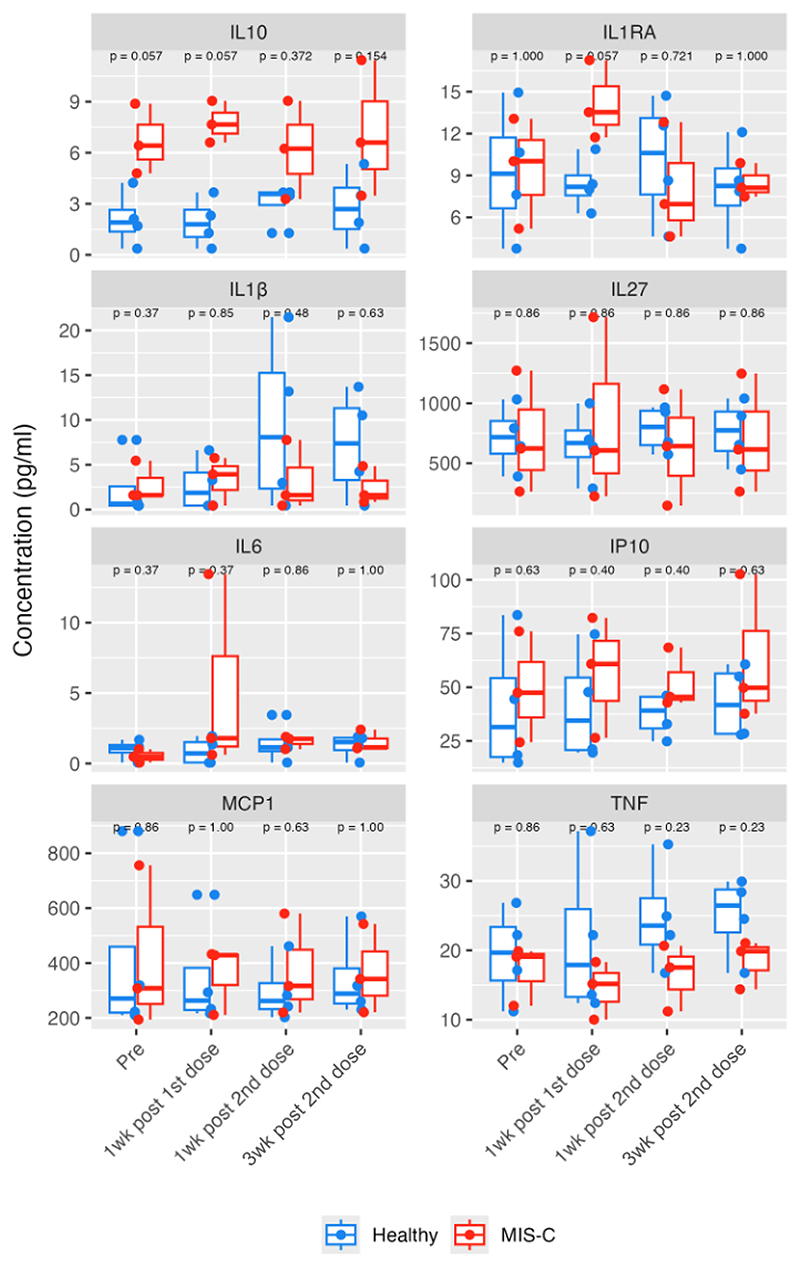
Serum cytokine concentration in participants who have a history of MIS-C (red) and healthy participants (blue) throughout SARS-CoV-2 vaccination schedule.

At all four timepoints, there was no difference in the serum concentration of IL-1β, IL-1RA, IL-6, IL-27, IP-10, MCP-1 or TNF between children with a history of MIS-C and healthy children (Figure 3). At the pre-vaccine and one week post first dose sampling timepoints, children with a history of MIS-C had IL-10 serum concentrations that appeared higher compared to healthy controls (median concentration in children with previous MIS-C: 6.6 pg/ml; in healthy controls: 2.21 pg/ml; p = 0.057). However, these IL-10 concentrations were much lower than during acute MIS-C (median: 131.24 pg/ml). They were comparable to IL-10 serum concentrations from samples taken two weeks after MIS-C discharge (median: 5.43 pg/ml) and were within the normal range for the total cohort of healthy children (Supplementary Figure S1). Similarly, IL-1RA appeared slightly raised in children with a history of MIS-C at the second timepoint (median concentration in children with previous MIS-C: 13.53 pg/ml; in healthy controls: 8.19 pg/ml), although this was not significant (p = 0.057) and was lower than the acute phase of disease (median: 493.9 pg/ml). The serum concentrations of all measured cytokines were not affected by vaccination.

### Gene expression profiling reveals no differences in vaccine responses between children with previous MIS-C and healthy children

**Figure 4:**
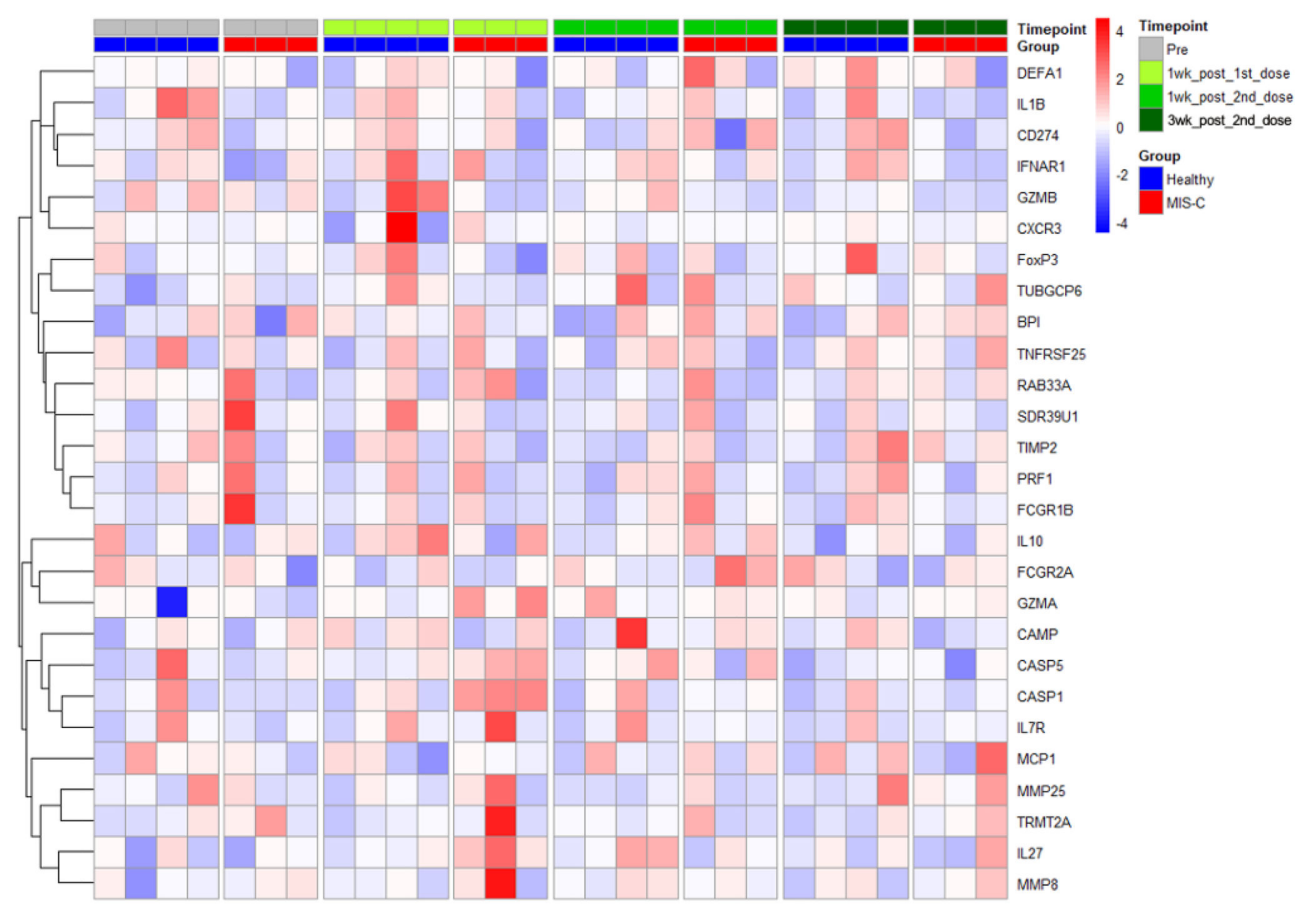
Hierarchical clustering of expression of gene transcripts in whole blood. Samples are grouped by type (vaccinated MIS-C patients and vaccinated healthy controls) and longitudinally (four vaccine visits corresponding to pre-vaccination, first dose, second dose and final follow-up). Clustering is supervised by timepoint and group.

Hierarchical clustering of gene transcripts, supervised by type and visit, did not indicate any clear distinctions in gene expression between the groups (Figure 4). The most noticeable differences occurred after the first dose, with *CASP1* (p = 0.057) and *IL27* (p = 0.057) up-regulated in children with previous MIS-C compared to vaccinated healthy children and *TUBGCP6* (p = 0.057) down-regulated in these patients at this timepoint (Supplementary Figure S2). Expression of these transcripts was not significantly different when compared to 74 unvaccinated healthy children. There were no significant differences in gene expression between the groups at baseline or at the final follow-up three weeks after the second dose.

When comparing to acute MIS-C, we observed a significant distinction between unvaccinated acute MIS-C and unvaccinated healthy controls (Supplementary Figure S3). Gene expression from participants who received the SARS-CoV-2 vaccine clustered almost entirely with the healthy control participants at all timepoints regardless of previous MIS-C (Supplementary Figure S3). Further, PCA of all transcripts revealed separate clusters of acute MIS-C and healthy controls, with vaccinated participants clustering with healthy individuals (Supplementary Figure S4).

### T cell Vβ21.3 expression and SARS-CoV-2 specific responses

**Figure 5:**
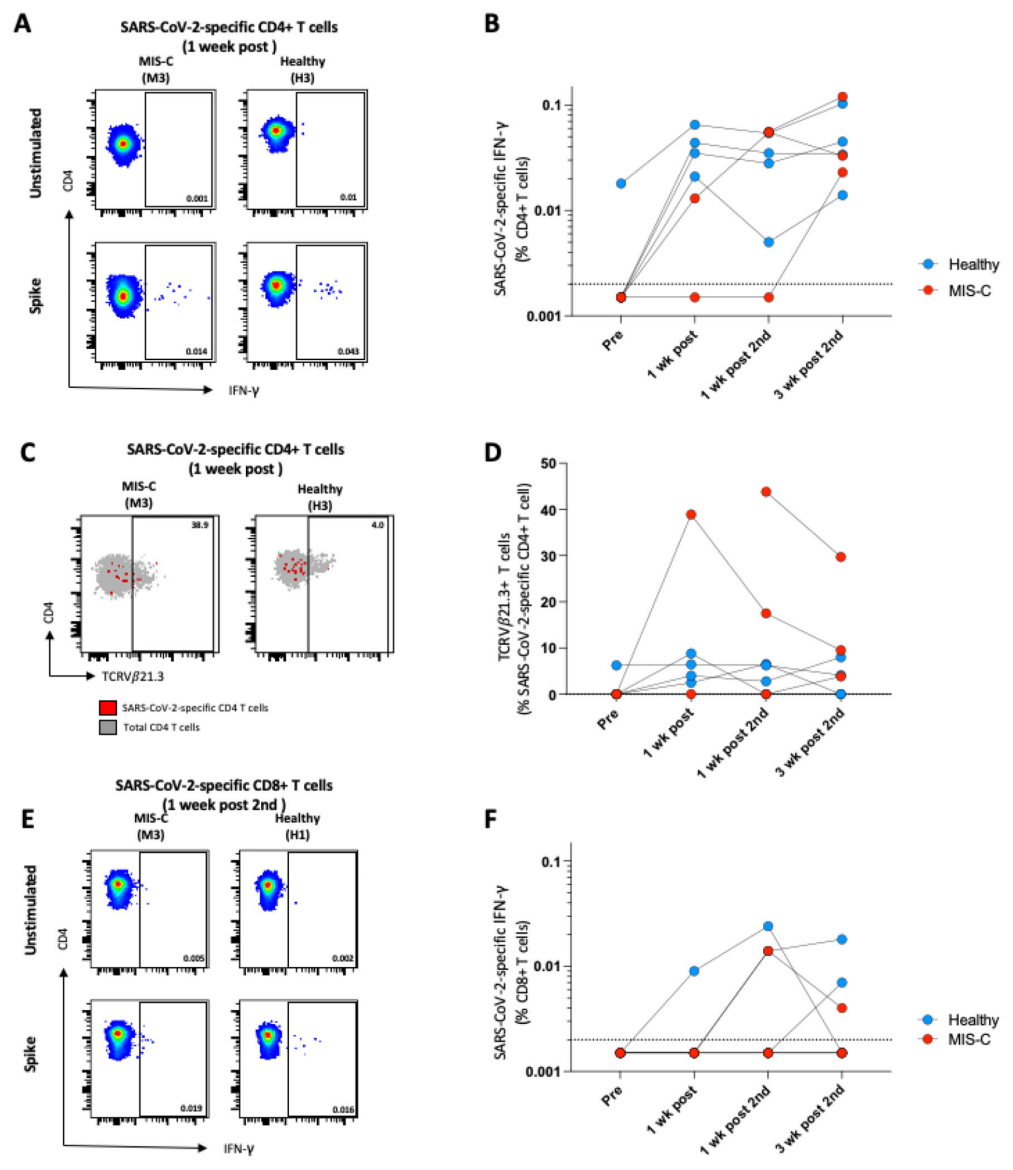
SARS-CoV-2-specific CD4 T cell responses in vaccine recipients. (A) Representative flow cytometry plots showing IFN-γ production from whole blood stimulated with spike peptides or the unstimulated control. (B) The frequency of SARS-CoV-2-specific CD4+IFN-γ+ T cells in participants with a history of MIS-C (red), or healthy participants (blue) before receiving a dose of the SARS-CoV-2 vaccine, one week after the first dose of the vaccine, one week after the second dose of the vaccine and three weeks after the second dose of the vaccine. (C) Overlay flow plot examples of TCR Vβ21.3 profile in SARS-CoV-2-specific CD4 T cells in a healthy (left) or MIS-C (right) child. (D) The percentage of SARS-CoV-2-specific CD4 T cells expressing TCR Vβ21.3 across the vaccination schedule. A minimum of 15 CD4+IFN-γ+ events were required for phenotyping. (E) Representative CD4 T cell flow cytometry plots showing IFN-γ production. (F) The frequency of SARS-CoV-2-specific CD8+IFN-γ+ T cells. One of the participants who previously had MIS-C only had samples available from after the second dose. Note that some individual values may be very similar: for individual datapoints please refer to Supplementary Table S3.

T cell analysis was performed on all participants’ samples at all timepoints, except for one participant (M2) for whom T cell analysis could only be performed at the final two timepoints (Figure 5). Prior to vaccination only 1/4 (25%) of the healthy children and no children with a history of MIS-C had a detectable SARS-CoV-2-specific CD4 T cell response (Figure 5B). After the first SARS-CoV-2 vaccination, 4/4 healthy children had a detectable SARS-CoV-2-specific CD4 T cell IFN-γ response, which was maintained at one week post second vaccination and three weeks after the second dose (Figure 5B). Of the children with a history of MIS-C, one child (M3) gained a T cell response following the first vaccination which was boosted one week post the second vaccination (median: 0.013% vs 0.055% after the first and second vaccination respectively). The second child with a history of MIS-C (M1) gained a response after two vaccination doses at the final timepoint, while the third child for which we had no pre-vaccination data (M2), had a SARS-CoV-2-specific CD4 T cell IFN-γ response at both the one-week post second vaccination and final timepoints. There were no significant differences in the magnitude of the SARS-CoV-2-specific CD4 T cell IFN-γ responses between the healthy children and those with a history of MIS-C, with all children gaining robust CD4 T cell responses by three weeks after the second dose (Figure 5B). Fewer participants had SARS-CoV-2-specific CD8 T cell IFN-γ responses (Figure 5E and F). One of the healthy children had a detectable CD8 T cell IFN-γ response after the first vaccination and two had a response after the second vaccination, while only one child with a history of MIS-C developed a SARS-CoV-2-specific CD8 T cell IFN-γ response. This was detectable at one week after the second vaccination and the final timepoint (Figure 5E and F).

We next examined CD4 and CD8 T cell expression of TCR Vβ21.3, due to its reported expansion in patients with MIS-C [10]. There was no difference in the frequency of total CD4 or CD8 T cells expressing TCR Vβ21.3 between the groups at any time timepoint (Supplementary Figure S5). There was also no significant change in the frequency of CD4 or CD8 Vβ21.3+ T cells between the pre-vaccination timepoint and a week after the second dose, either for children with previous MIS-C or for healthy children. We next characterised the SARS-CoV-2-specific CD4 T cells and there was a transient increase in the proportion of SARS-CoV-2-specific CD4 T cells expressing TCR Vβ21.3 in children with a history of MIS-C relative to vaccinated healthy children. In participant M3, 38.9% of SARS-CoV-2-specific T cells expressed TCR Vβ21.3 at the one-week timepoint which decreased to 17.5% and 9.5% at the one-week post second vaccine and six-week timepoints respectively. Although there was a lack of pre-vaccination data, participant M2 also had high proportion of TCR Vβ21.3 expression (43.8%) one week after the second vaccine dose, decreasing to 29.7% at the six-week timepoint. In contrast, TCR Vβ21.3 expression on the SARS-CoV-2-specific T cells in healthy children remained below 10% at all timepoints (Figure 5C and D).

## Discussion

MIS-C is a poorly understood acute immune response to SARS-CoV-2 exposure. Although SARS-CoV-2 vaccination has been shown to be safe in children with previous MIS-C [16–20], the effect of antigen re-exposure on cytokine expression, gene expression and T cell responses is not known. In this study we confirm that SARS-CoV-2 vaccination in children with previous MIS-C results in robust SARS-CoV-2-specific antibody and T cell responses, with comparable viral neutralisation capacity to vaccinated children with no history of MIS-C. These immune responses were observed following the first SARS-CoV-2 vaccine dose and were maintained through the second dose and to the end of the follow-up period, three weeks after the second dose. SARS-CoV-2 vaccination did not induce inflammatory cytokine or gene expression responses and did not result in an increased proportion of CD4 or CD8 TCR Vβ21.3+ T cells in children with previous MIS-C compared to those without. Intriguingly however, this small series showed a transient increase in the proportion of SARS-CoV-2-specific T cells expressing TCR Vβ21.3 in children with a history of MIS-C relative to vaccinated healthy children.

Despite initial concerns that SARS-CoV-2 vaccination could cause MIS-C recurrence, and case reports of MIS-C onset after vaccination [27–37], larger-scale cohort studies and pharmacovigilance studies have shown this to be a very rare occurrence indeed [15, 38], more likely due to breakthrough infection after SARS-CoV-2 exposure than an immunological response to the vaccine itself. We showed a boost in spike IgG antibodies and neutralisation ability in patients with and without previous MIS-C shortly after vaccination and an average of 18 days after the vaccine course was concluded. These findings are in accordance with studies from other regions in the USA and Europe. A prospective cohort study of 20 Polish children with prior MIS-C demonstrated the immunological benefit of the Pfizer-BioNTech mRNA vaccine, with children mounting effective spike IgG antibodies a median of 23 days after completing the vaccine course [39]. There was also no difference in IgG responses between these children and 34 healthy children vaccinated at the same time [39]. Vaccination boosted spike IgG antibodies for up to three months in a series of seven children with previous MIS-C [40], with similar neutralising ability and cytotoxicity as in a cohort of vaccinated healthy children. What appeared to influence neutralising antibodies more was the infecting SARS-CoV-2 variant, with neutralising titres significantly reduced for the Omicron variant (B.1.1.529) compared to Wild-type [40]. In two small case series totalling five children with previous MIS-C, SARS-CoV-2 vaccination led to elevation of IgG antibodies to levels higher than in acute MIS-C [41, 42].

Through longitudinal profiling of participants’ immunological status, we provide further evidence of the overall tolerance of children to vaccination. As a hyperinflammatory condition, MIS-C is characterised by elevation of numerous inflammatory cytokines and markers [1–8]. In our setting, we previously found elevated IL-10 and IL-6 to be specific to MIS-C compared to other clinically similar acute inflammatory diseases [1]. In the present study, inflammatory cytokine levels were low in both healthy children and those with previous MIS-C at the start of the vaccination programme, with no observed clinical effect of vaccination on the production of any of the tested cytokines. Notably, IL-10 appeared elevated in children with prior MIS-C at two timepoints, including before vaccination, although the observed levels were within the normal range and much lower than during acute MIS-C [1]. Similarly, gene expression profiling revealed no differences between the experimental groups, and transcript-level vaccine responses were virtually indistinguishable between patients with prior MIS-C and healthy children. This is in contrast to the widespread levels of gene dysregulation we have described in acute MIS-C [24].

All of the children, regardless of previous MIS-C, gained a SARS-CoV-2-specific CD4 T cell response during the course of the study. Despite positive anti-spike titres in all participants, it is notable that only one, a healthy child, had detectable T cell responses before vaccination. We have previously shown lack of T cell responses in 17% of seropositive children in our setting [43]. Here, most children mounted a T cell response after the first dose, with one of the children with previous MIS-C only mounting a T cell response after the second vaccination. Nevertheless, at the last timepoint, there was no difference in the magnitude of T cell responses between the two groups. Polyclonal TCR Vβ21.3 T cell expansion is characteristic of MIS-C and not observed in COVID-19 or other similar inflammatory conditions [10, 44], and suggestive of a superantigen response to SARS-CoV-2 in MIS-C. Interestingly, a transient increase of the proportion of SARS-CoV-2-responding CD4 T cells expressing TCR Vβ21.3 was observed in two of the children with previous MIS-C (66.7%) after the first vaccine dose, while this was not observed in any of the healthy children. While a superantigen-like polyclonal TCR Vβ21.3 T cell expansion induced by spike peptide stimulation could possibly contain not only SARS-CoV-2-specific T cells, these IFN-γ producing T cells do represent a subset of activated T cells within which to analyse TCR Vβ21.3 expression. Studies have shown an increased proportion of activated T cells expressing Vβ21.3 during MIS-C disease [10, 45].

This study was limited by the small sample size, which makes it underpowered to detect significant differences between the experimental groups and to draw definitive conclusions. The exclusion of participants younger than 12 years was due to South African regulations at the time of study design which had not yet authorised paediatric vaccinations at ages younger than this. Another limitation is that data concerning safety and adverse events were not collected as this was beyond the scope of the investigation. Nevertheless, our use of in-depth profiling provides some of the first data on inflammatory responses to SARS-CoV-2 vaccination in children with and without previous MIS-C.

A reduction in MIS-C incidence has been observed across the globe and is thought to be due, at least in part, to SARS-CoV-2 vaccination [46]. Nationwide cohort studies have demonstrated that vaccination reduces the risk of MIS-C in children and adolescents [12–14, 47–52]. We still do not fully understand the underlying causes of this disease. These data describing the immune responses upon SARS-CoV-2 vaccination add to the global understanding of this enigmatic disease. Although larger-scale cohort studies and randomised clinical trials are needed to confirm these findings, the present evidence further supports SARS-CoV-2 vaccination in children with previous MIS-C.

## Supplementary Material

Supplementary Figure S1
