# Supplementary material for: Humoral, T cell and immune gene expression responses to SARS-CoV-2 vaccination in a small group of children with previous MIS-C
Source: Vaccine. Author manuscript; Available in PMC 2025 Sep 10. (PMC7618102; doi:10.1016/j.vaccine.2025.127461)
Supplement: Supplementary Figure S1 [file EMS208396-supplement-Supplementary_Figure_S1.pdf]

Supplementary material

Supplementary Table S1

Table S1: Summary demographics of vaccinated participants

	**Children with previous MIS-C (n = 3)**	**Healthy children (n = 4)**
Median age at first sample	12.43	12.37
Female (%)	1 (33%)	2 (50%)

Supplementary Table S2

Table S2: Admission summary of vaccinated children with previous MIS-C

	**Patient M1**	**Patient M2**	**Patient M3**
Date of admission	19 June 2020	4 January 2021	4 January 2021
Age at admission (years)	10	12	11
Duration of stay (days)	10	11	8
ICU	-	+	+
SARS-CoV-2 PCR	Negative	Positive	Positive
SARS-CoV-2 antibody	Positive	Positive	Positive
Fever	+	+	+
Rash	-	+	+
Tachycardia	+	+	+
Conjunctivitis	-	+	-
Mucositis	-	+	-
Hypotension	-	+	+
Diarrhoea	-	+	+
Abdominal pain	+	+	+
Arthritis	-	+	-
Lung disease	+	+	+
CNS disease	-	+	-
Maximum CRP (mg/L)	357	511	300
Minimum platelets ($\times10^9$/L)	193	194	91
Minimum sodium (mmol/L)	131	128	129
Maximum neutrophils ($\times10^9$/L)	5.56	21.18	23.99
Minimum lymphocytes ($\times10^9$/L)	1.04	0.75	0.13
Maximum Trop-T (ng/L)	41	94	ND
Maximum pro-BNP (ng/L)	6,044	>35,000	10,148
IVIG dose (mg/kg)	2	2	1
Methylprednisolone dose (mg/kg)	-	30	30
Ejection fraction (%)	30	65	50
Meets Kawasaki Disease criteria	No	Yes	No

Supplementary Table S3

Table S3: Individual datapoints for the serum anti-SARS-CoV-2 antibody assays and CD4 T cell responses

Measure (units)*	Patient M1	Patient M2	Patient M3	Control H1	Control H2	Control H3	Control H4
Spike IgG (OD450) (Figure 2A)							
Timepoint 1	2.976	2.378	3.142	3.204	2.432	2.935	2.338
Timepoint 2	3.450	3.489	3.417	3.508	3.515	3.467	3.322
Timepoint 3	3.615	3.708	3.549	3.507	3.362	3.390	3.609
Timepoint 4	3.428	3.557	3.449	3.396	3.357	3.426	3.372
Neutralising ability (ID_{50}) (Figure 2B)							
Timepoint 1	1071	186	1389	505	32	1038	801
Timepoint 2	8144	9942	4377	26886	3939	4323	22871
Timepoint 3	14345	55031	20368	20210	8178	37371	24732
Timepoint 4	26011	31246	58861	88470	8962	26131	43818
SARS-CoV-2-specific IFN-γ (% CD4+ T cells) (Figure 5B)#							
Timepoint 1	0	NR	0	0	0	0	0.018
Timepoint 2	0	NR	0.013	0.021	0.044	0.035	0.065
Timepoint 3	0	0.056	0.055	0.005	0.035	0.028	0.054
Timepoint 4	0.23	0.12	0.033	0.014	0.034	0.045	0.103
SARS-CoV-2-specific IFN-γ (% CD8+ T cells) (Figure 5F)#							
Timepoint 1	0	NR	0	0	0	0	0
Timepoint 2	0	NR	0	0	0	0.009	0
Timepoint 3	0	0	0.014	0.014	0	0.024	0
Timepoint 4	0	0	0.004	0.018	0.007	0.063	0
TCRV*β*21.3+ T cells (% SARS-CoV-2-specific CD4+ T cell) (Figure 5D)							
Timepoint 1	0	NR	0	0	0	0	6.25
Timepoint 2	0	NR	38.9	8.82	2.5	4	6.45
Timepoint 3	0	43.8	17.5	0	6.52	2.78	6.25
Timepoint 4	3.85	29.7	9.52	0	0	8	4.11
Frequency of TCRV*β*21.3+ CD4+ T cells (%) (Figure S5B)							
Timepoint 1	0.951	NR	1.679	2.667	1.475	1.71	2.742
Timepoint 2	1.207	NR	1.815	2.136	1.896	1.871	2.054
Timepoint 3	1.906	2.557	2.105	3.351	1.668	1.82	2.707

Timepoint 4	2.093	2.335	2.201	2.521	2.016	2.214	2.627
Frequency of TCRVβ21.3+ CD8+ T cells (%) (Figure S5C)							
Timepoint 1	0.311	NR	0.822	0.852	0.54	0.981	2.084
Timepoint 2	0.469	NR	1.14	0.82	1.114	1.49	1.587
Timepoint 3	1	1.86	1.763	1.493	0.577	1.136	2.115
Timepoint 4	1.081	1.187	2.28	1.325	0.948	1.665	2.105

NR, not recorded

* Timepoints 1: pre-vaccination; Timepoint 2: one week after the first dose; Timepoint 3: one week after the second dose; Timepoint 4: six weeks after the first dose

[#] Non-responders (0s) were assigned an arbitrary value of 0.0015 for the purposes of graphing on log scales

Supplementary Figure S1

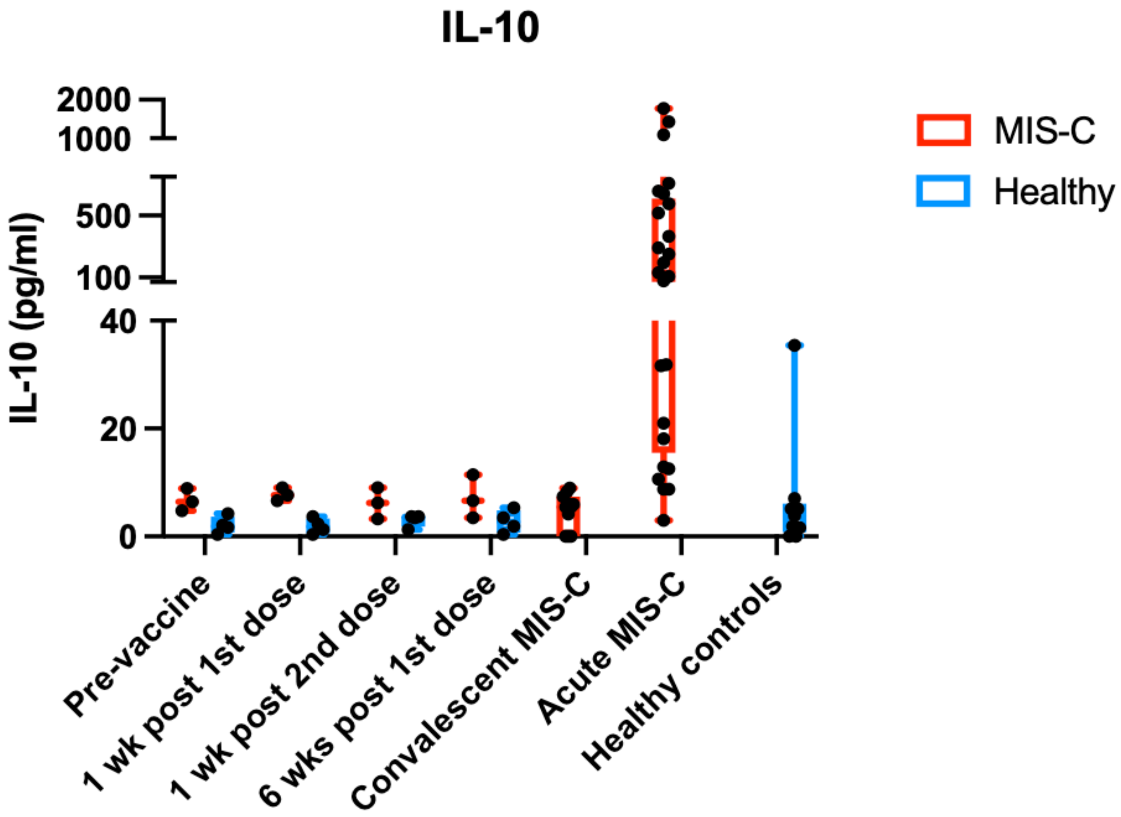

Supplementary Figure S1: IL-10 serum concentrations in vaccinated children, children with acute and convalescent MIS-C, and healthy unvaccinated children.

Supplementary Figure S2

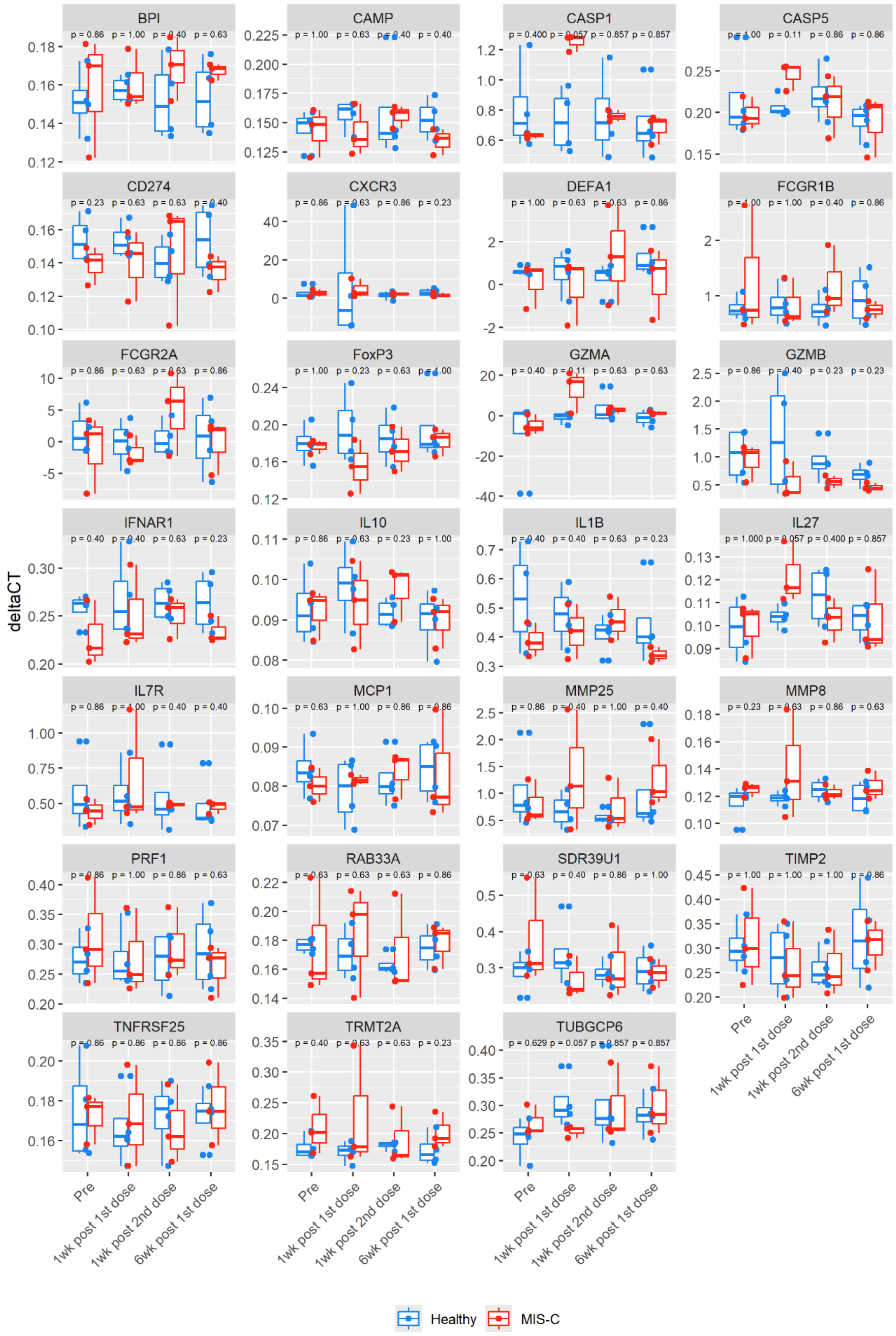

Supplementary Figure S2: Gene expression in participants who have a history of MIS-C (red) and healthy participants (blue) throughout SARS-CoV-2 vaccination schedule.

Supplementary Figure S3

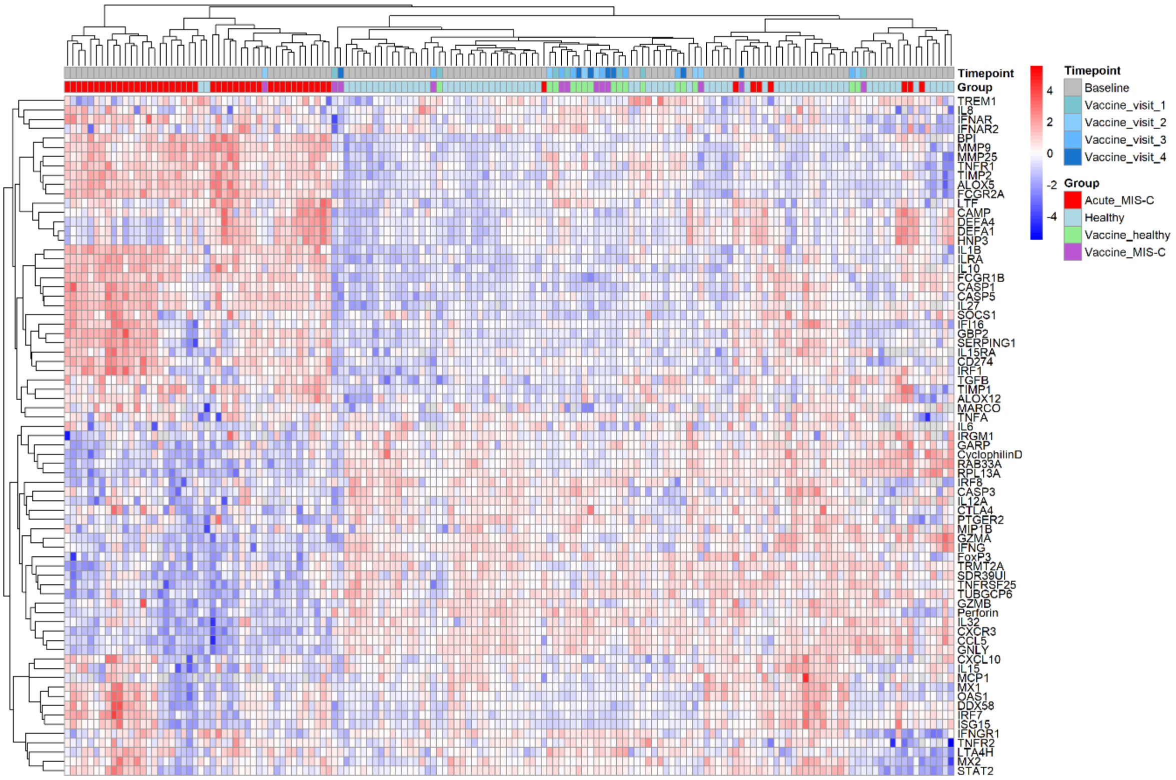

Supplementary Figure S3: Unsupervised hierarchical clustering of expression of all transcripts. Samples are grouped by type (unvaccinated acute MIS-C, unvaccinated healthy controls, vaccinated MIS-C patients and vaccinated healthy controls) and longitudinally in the case of vaccinated participants (baseline and three follow-up visits).

Supplementary Figure S4

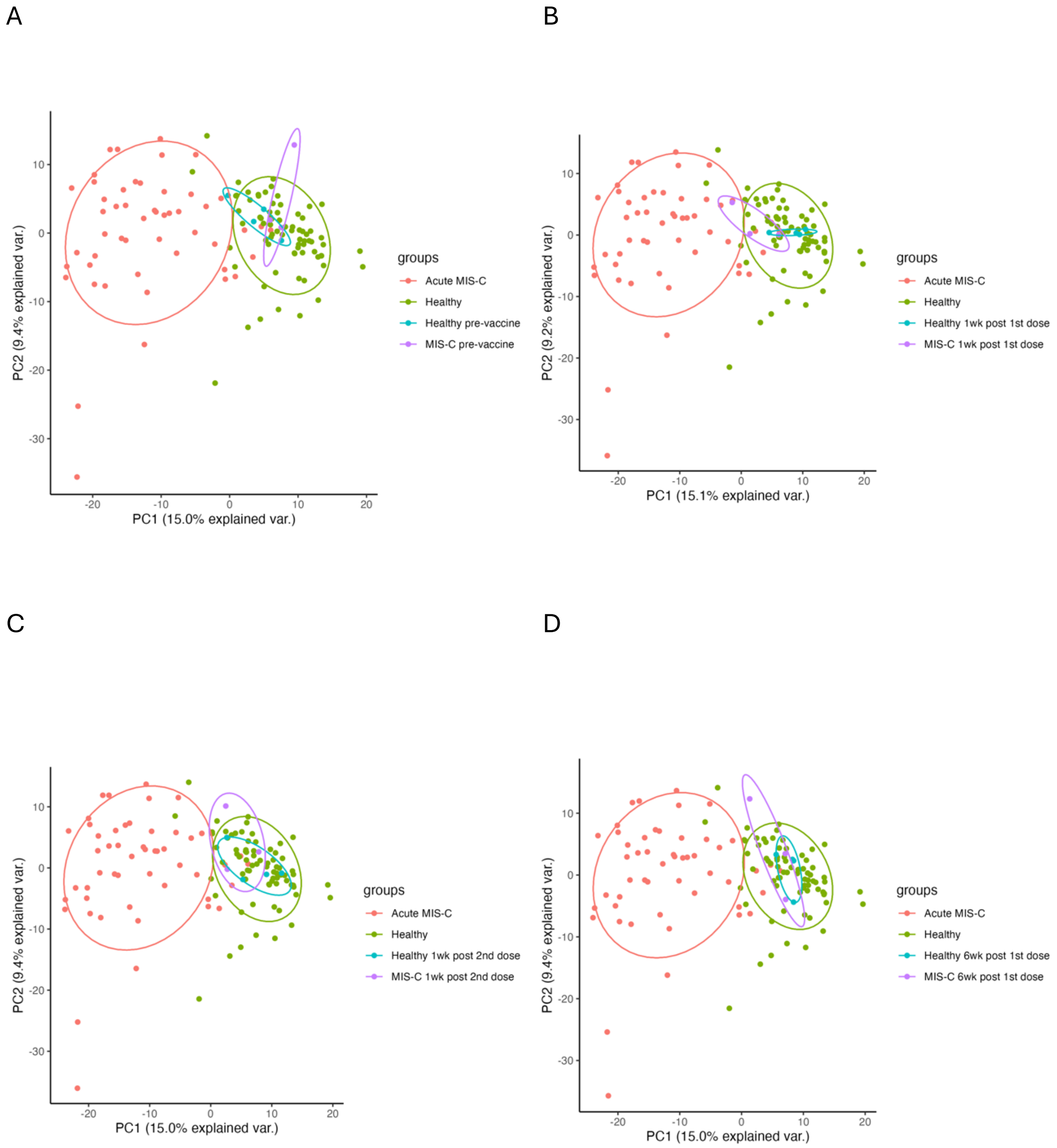

Supplementary Figure S4: Principal component analysis of gene expression in healthy controls, acute MIS-C at baseline, vaccinated MIS-C patients and vaccinated healthy children at the study timepoints: (A) pre-vaccination; (B) one week after the first dose; (C) one week after the second dose; and (D) six weeks after the first dose. Plotted are principal components 1 and 2.

Supplementary Figure S5

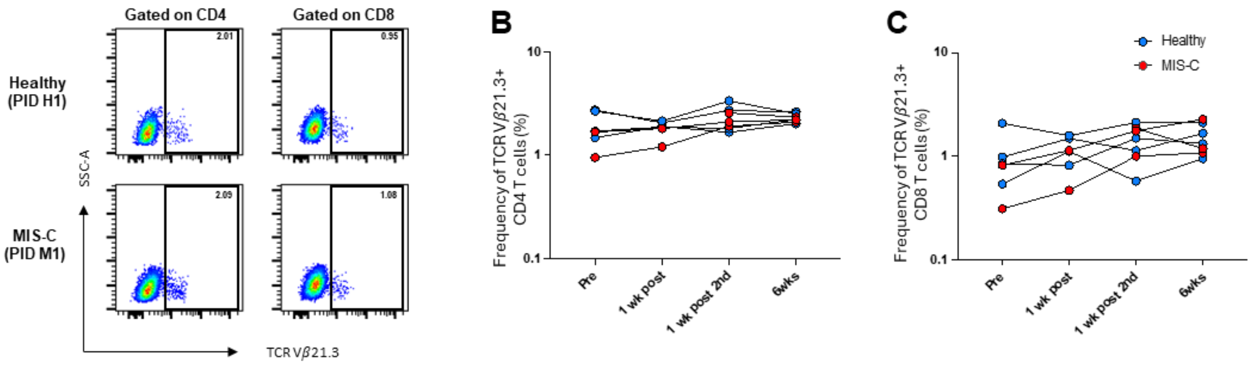

Supplementary Figure S5: Vβ21.3 expression on CD4 and CD8 T cells. (A) Representative examples of CD4 (left) and CD8 (right) T cell expression of TCR Vβ21.3 in a previously healthy child (top row) or child with prior MIS-C (bottom row). Frequency of CD4 (B) and CD8 (C) T cells expressing TCR Vβ21.3 in participants with a history of MIS-C (red), or healthy participants (blue) before receiving a dose of the SARS-CoV-2 vaccine, one week after the first dose of the vaccine, one week after the second dose of the vaccine and six weeks after the first dose of the vaccine.
